# Sleeplessness

**Published:** 1871-11

**Authors:** 


### SLEEPLESSNESS.

THE best anodyne is a liberal amount of muscular activity out of doors every day. Persons who sit around the fire and lounge on the sofa, or read or sew a great part of the day, need not expect sound sleep ; only the laboring man can taste it in all its sweetness.

Many fail to sleep at night because they will persist in sleeping in the day-time. It is just as impossible to healthfully force more sleep on the system than the proportion of exercise requires, as to force the stomach to digest more food than the body requires. Rather than court sleep by industrious activities, many persons resort to medicine, and every new drug which is heralded as a promoter of sleep becomes at once immensely popular, even though it is known to possess dangerous qualities.

CHLORAL HYDRATE

has had a great run, and even young men are known to be purchasing it at the drug stores, to be used in promoting sleep; it should never be taken unless advised by the family physician, for the medical journals are constantly publishing cases where serious harm and even fatal results attend its habitual use. A lady, aged fifty, could not sleep on account of some heart sorrow, and took chloral in thirty grain doses, and, taking it nightly, she died in four or five days, as an apoplectic person. The brain, on examination, was very deeply congested; so full of blood as to cause a cessation of all the functions of life.

The disposition of people to swallow medicine for every little trifling thing is amazing, and that they purchase almost anything that is advertised is proven by the fact that so much advertising is done in that way; in the morning paper of to-day, a druggist states that he has spent, within a few months, one hundred and thirty thousand dollars in advertising a new medicine; and no doubt it has quite as much virtue as a pill of moonshine.

